# Acute Pyogenic Osteomyelitis of the Pubic Bone in a Patient with Systemic Lupus Erythematosus Mimicking Fracture of the Pubic Bone and Periprosthetic Joint Infection of the Hip

**DOI:** 10.1155/2021/6665938

**Published:** 2021-06-20

**Authors:** Aysha Rajeev, Jason Mavrotas, Sanjay Taribagil, Jonathan Loughead

**Affiliations:** Queen Elizabeth Hospital, Gateshead Health Foundation NHS Trust, Sheriff Hill, Gateshead NE9 6SX, UK

## Abstract

**Introduction:**

Pubic bone osteomyelitis is atypical, and the diagnosis is often overlooked. It may present as osteitis pubis, fracture, or noninfectious inflammation of the pubic symphysis. *Case Report*. We report a case of a 65-year-old lady who has systemic lupus erythematosus with acute pyogenic osteomyelitis of the pubic bone who presented initially with a suspicious healing pubic rami fracture and periprosthetic infection of the hip joint.

**Conclusion:**

Acute osteomyelitis of the pubic bone has often an infrequent and delayed presentation. Clinical awareness, early diagnosis, and appropriate treatment including surgical debridement and long-term antibiotics can prevent ongoing morbidities such as chronic osteomyelitis, pain, and deformities of pelvic bone and joints.

## 1. Introduction

Osteomyelitis of the pubic bone is a rare clinical condition and accounts for 2% of all haematogenous osteomyelitis in bone [1, 2]. Risk factors include intravenous drug use, pelvic surgery such as urological or obstetric, immunosuppression, and being an athlete [3, 4]. The most common organism causing infection is Staphylococcus aureus, with Pseudomonas aeruginosa seen most commonly in intravenous drug users [5]. We describe a case of acute pyogenic osteomyelitis of the pubic bone, caused by Escherichia coli (E.coli), presenting as a periprosthetic infection of the hip in a patient on long-term immunosuppression for systemic lupus erythematosus (SLE).

## 2. Case Report

A 65-year-old female was admitted with a one-week history of general malaise, decreased appetite, fever, lethargy, and progressively worsening pain in her right hip and groin. She had a history of a fall 4 weeks prior to presentation but had been mobilised with some discomfort following the fall. Past medical and surgical history included SLE, managed through regular corticosteroids and intermittent courses of hydroxychloroquine, and bilateral hip and knee replacements. On examination, she was unable to weight bear on the right lower limb. She had tachycardia and a temperature of 38.5°. There was severe tenderness to the right groin, a restricted range of movement, and a 20° fixed flexion deformity. The examination of the left hip and both knees was unremarkable.

**Figure 1:**
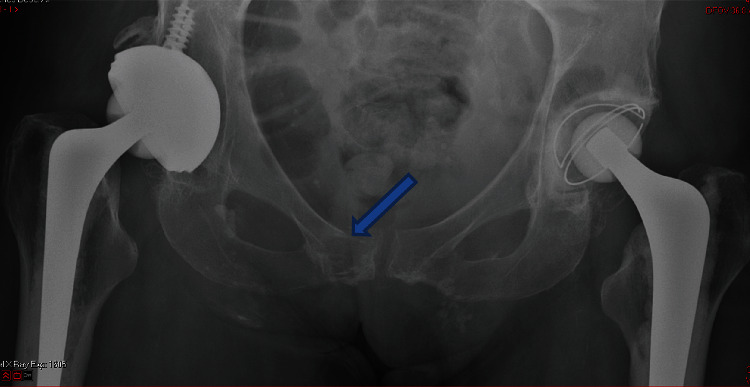
Plain radiograph showing irregularity in the left pubic bone and new bone formation.

Inflammatory markers were raised with WCC 19.83 and a raised CRP of 215. Plain radiographs of the pelvis showed bilateral total hip replacements in situ and an irregularity of the cortex of the superior pubic rami with evidence of new bone formation (Figure 1). A provisional clinical diagnosis of a periprosthetic infection of the right hip was made, with an ipsilateral pubic ramus fracture.

**Figure 2:**
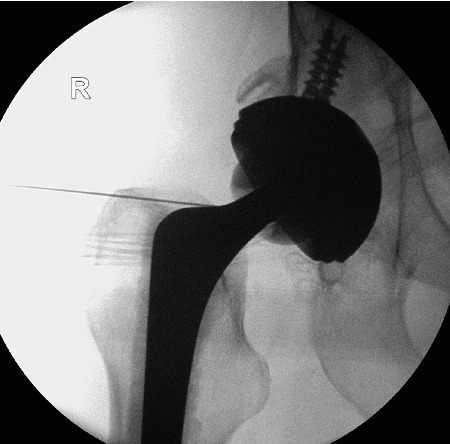
Image intensifier picture showing aspiration of the total hip replacement.

**Figure 3:**
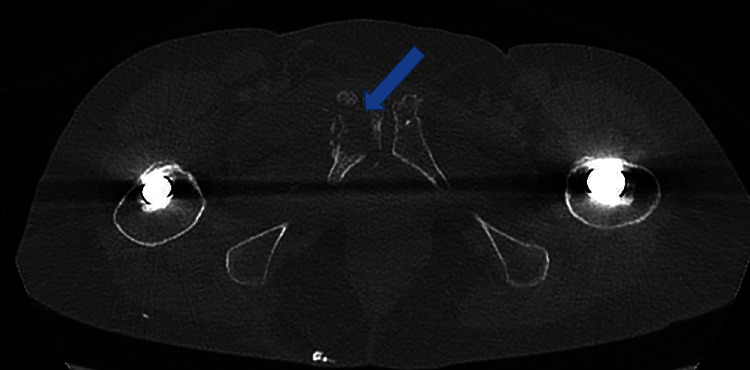
CT scan showing destruction of pubic bone and fluid collection suggesting acute osteomyelitis.

Arthrocentesis of the right hip under the image intensifier was dry (Figure 2). A contrast CT scan of the pelvis showed a lytic area at the right pubic bone with fragmentation of the bone and expansion of the soft tissues with abnormality of the upper right adductors. Fluid collections were noted in the expansion of the adjacent muscles, consistent with abscess formation secondary to osteomyelitis (Figure 3).

The patient underwent exploration of the right pubic region under general anaesthesia by a joint team of orthopaedic and general surgeons. Exploration revealed abscess formation around the right pubic bone with destruction of the cortex of the superior pubic ramus and evidence of osteomyelitis and the collection extending into the adductor compartment. The pus and tissue samples were sent for culture and sensitivity, identifying E.coli sensitive to cefuroxime. She was treated with intravenous cefuroxime for two weeks, later receiving a further four weeks of oral cefuroxime at the time of discharge from the hospital. An outpatient follow-up at three months indicated that inflammatory markers had settled, and she was mobilising pain-free. Repeat CT scan and inflammatory markers confirmed resolution of the osteomyelitis and persisting normalisation of her CRP.

## 3. Discussion

This case presents a uniquely difficult diagnostic challenge, requiring the discrimination between three distinct pathologies in an anatomically and functionally intertwined region: septic arthritis of the hip and fracture and osteomyelitis of the pubic bone. In all these pathologies, the patient can present with atraumatic hip or groin pain that is insidious in onset, unremitting, and exacerbated by movement. Features of septic shock with leucocytosis are suggestive of an infective pathology, namely, septic arthritis of the hip or osteomyelitis.

Differentiating between septic arthritis of the hip and osteomyelitis of the pubic bone is inherently difficult. Appropriate imaging has a fundamental role in the diagnosis of osteomyelitis. In our case, early plain radiographs indicated an irregularity of the cortex of the superior pubic rami with evidence of new bone formation and radiolucency. While affordable and quick, plain radiographs have low sensitivity and specificity for detecting osteomyelitis with as many as 80% of patients showing no radiographic changes within the first two weeks of infection [6]. Typical early changes include periosteal thickening, lytic lesions, osteopenia, loss of trabecular architecture, and new bone apposition [7].

MRI imaging remains the gold standard for investigating osteomyelitis. It is highly sensitive for osteomyelitis due to its ability to detect bone marrow oedema, detecting infection as early as one to two days after the onset [6]. In our case, CT imaging was used due to the clinical instability of the patient and an out-of-hour requirement for imaging. CT imaging is limited by its inability to detect bone marrow oedema, and furthermore, a normal CT cannot exclude early osteomyelitis. It does, however, provide excellent delineation of osseous changes making it superior to MRI for the detection of sequestra, cloaca, involucra, or intraosseous gas and is frequently used to obtain image-guided biopsies [8].

The precise aetiology of our patient's presentation still remains unclear. The mechanisms of action of glucocorticosteroids consist of genomic and nongenomic pathways triggered following the activation of intracellular glucocorticoid receptors. These result in a breakdown of the inflammatory cascade through reduction of T and B cell proliferation, immunoglobulin and cytokine synthesis, and inhibition of mononuclear cell and neutrophil leucocyte migration [9]. Consequently, it increases the risk of opportunistic infections by bacteria, fungi, and viruses and thus may in part serve to explain the rare presentation of osteomyelitis seen here.

The immunomodulatory properties of hydroxychloroquine have also been well researched [10]; however, it is unclear whether the intermittent courses highlighted in the case have contributed to the overall risk of opportunistic infection. In a study of 3030 patients, patients starting prednisolone doses ≤ 15 mg/day without antimalarial had a fourfold greater risk of serious infection than did patients with SLE starting antimalarial only [11]. The processes affected include chemotaxis, production of interleukin 8 (IL-8), oxidative metabolism, membrane recognition, phagocytosis, and the complement system [12]. Thus, SLE, both through its pathogenesis and its treatments, represents a significant risk factor for serious infections, a 6-7-fold greater risk according to a study [11].

SLE has been shown to predispose patients to osteonecrosis. Intraosseous adipocyte hypertrophy, fat conversion of red marrow, and subsequent raised bone marrow pressure have been hypothesised as a mechanism with glucocorticoids causing these [13]. A role for antiphospholipid syndrome in osteonecrosis and stress fractures has also been postulated, thus providing a glucocorticoid-independent risk factor. Here, antiphospholipid antibodies are thought to induce a hypercoagulable state that can lead to vessel thrombosis, bone ischaemia, and necrosis [14]. Osteonecrosis caused directly or indirectly by SLE and its therapies can provide the foundation for opportunistic, haematogenous E.coli pelvic osteomyelitis. Minassian et al. reported seven cases of pubic bone osteomyelitis complicating radiotherapy-induced osteonecrosis [15].

## 4. Conclusions

Osteomyelitis of the pubic bone is a rare but serious life-threatening condition. It can mimic traumatic or stress fracture of the pubic bone and septic arthritis of the hip. Early diagnosis with either MRI or CT scan is indicated. Treatment includes debridement and long-term antibiotics. Patients with autoimmune diseases such as SLE on glucocorticoids and other immunomodulators are at risk of opportune osteomyelitis.

## Data Availability

The data used to support the findings of this study are included within the article.
